# Detection and Molecular Characterization of Circulating Tumour Cells: Challenges for the Clinical Setting

**DOI:** 10.3390/cancers15072185

**Published:** 2023-04-06

**Authors:** Areti Strati, Athina Markou, Evgenia Kyriakopoulou, Evi Lianidou

**Affiliations:** 1 Analysis of Circulating Tumour Cells Lab, Laboratory of Analytical Chemistry, Department of Chemistry, National and Kapodistrian University of Athens, 15771 Athens, Greece; astrati@chem.uoa.gr (A.S.); atmarkou@chem.uoa.gr (A.M.); 2 St. Barthlomew’s Hospital, Barts Health NHS Trust, London EC1A 7BE, UK; kyriakopoulou.evgenia@gmail.com

**Keywords:** liquid biopsy, circulating tumour cells, circulating tumour DNA, single-cell analysis, breast cancer, prostate cancer, non-small-cell lung cancer

## Abstract

**Simple Summary:**

Liquid biopsy provides important information through the analysis of circulating tumour cells (CTCs) and circulating tumour-derived material, such as circulating tumour DNA (ctDNA), circulating miRNAs (cfmiRNAs) and extracellular vehicles (EVs). CTC analysis has already had an important impact on the prognosis, detection of minimal residual disease (MRD), treatment selection and monitoring of cancer patients. The purpose of this review is to summarize the latest findings on the clinical significance of CTCs for the management of breast, prostate and non-small-cell lung cancer patients.

**Abstract:**

Over the last decade, liquid biopsy has gained much attention as a powerful tool in personalized medicine since it enables monitoring cancer evolution and follow-up of cancer patients in real time. Through minimally invasive procedures, liquid biopsy provides important information through the analysis of circulating tumour cells (CTCs) and circulating tumour-derived material, such as circulating tumour DNA (ctDNA), circulating miRNAs (cfmiRNAs) and extracellular vehicles (EVs). CTC analysis has already had an important impact on the prognosis, detection of minimal residual disease (MRD), treatment selection and monitoring of cancer patients. Numerous clinical trials nowadays include a liquid biopsy arm. CTC analysis is now an exponentially expanding field in almost all types of solid cancers. Functional studies, mainly based on CTC-derived cell-lines and CTC-derived explants (CDx), provide important insights into the metastatic process. The purpose of this review is to summarize the latest findings on the clinical significance of CTCs for the management of cancer patients, covering the last four years. This review focuses on providing a comprehensive overview of CTC analysis in breast, prostate and non-small-cell lung cancer. The unique potential of CTC single-cell analysis for understanding metastasis biology, and the importance of quality control and standardization of methodologies used in this field, is also discussed.

## 1. Introduction

Liquid biopsy (LB), based on minimally invasive blood tests, provides an extremely powerful and reliable tool for the individual molecular profiling of patients in real time. In LB, important information can be derived through the isolation, enumeration and molecular analysis of circulating tumour cells (CTCs), circulating tumour DNA (ctDNA), circulating miRNAs, and tumour-derived extracellular vesicles (tdEVs) in various body fluids, mainly peripheral blood, but also urine, CSF, pleural fluid, and others. The identification of predictive biomarkers through LB in real time has strong potential for novel approaches in the therapeutic management of cancer patients [1]. LB is focused on early cancer detection, improved cancer staging, early detection of relapse, real-time monitoring of therapeutic efficacy, detection of therapeutic targets and resistance mechanisms. The FDA has approved several LB-based assays and, more recently, multigene assays to detect genetic alterations in plasma cell-free DNA (cfDNA) for use as companion diagnostic tests matched to specific molecularly targeted therapies for cancer [2].

Many technologies have been developed and validated for CTC enrichment, detection and molecular characterization [3]. The CellSearch^®^ system (Menarini Silicon Biosystems, Florence, Italy), was the first system for CTC detection and enumeration that received FDA approval in 2004; since then, it has been widely used in many clinical studies and for various types of cancer. This system is mainly based on the epithelial nature of CTCs, since CTC-enrichment is based on the expression of the epithelial marker EpCAM. However, it is now well known that CTCs are highly heterogeneous and that epithelial to mesenchymal transition (EMT) plays an important role in their isolation and enrichment strategies. Thus, a high variety of novel ‘label-free’ CTC enrichment technologies based on CTC size and deformability have been developed [4]. These technologies can be categorized as: (i) systems based on a combination of microfluidics and size-based enrichment, such as the Parsortix system, (Angle, UK), that was FDA-cleared in May 2022 for CTC enrichment in metastatic breast cancer (MBC), (ii) filtration devices based on different CTC size, such as the ScreenCell device (Screencell, Paris, France) and the ISET system (RareCells, France), (iii) microchips with integrated nanomaterials, such as the CTC chip (NP-HBCTC-Chip) and the graphene oxide (GO) chip, (iv) systems for single CTC isolation, such as the DepArray (Menarini, Silicon Biosystems, Italy), the VyCAP (VyCAP BV, The Netherlands), and the CellCollector (ALS, Sartorius), (v) epitope-agnostic systems that detect both EpCAM-pos and EpCAM-neg CTCs, such as the EPISPOT, and the Epic platform (Epic Sciences), (vi) density-based isolation followed by antibody cocktail for imaging (RareCyte system, Rarecyte, US), and (vii) molecular tests, such as the Adnatest (Qiagen, Germany), and many others [1,2,3,5,6,7,8,9,10,11].

**Figure 1:**
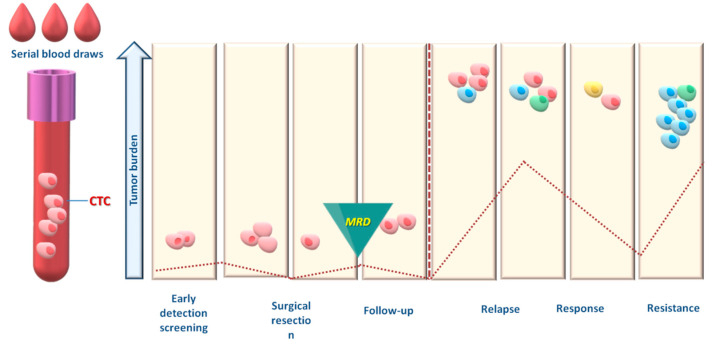
Applications of CTC analysis for the follow-up of cancer patients.

Up to now, most CTC studies were performed in patients with advanced-stage cancer, where CTC numbers are usually much higher than in early stage. The next goal for CTC analysis is the detection and characterization of minimal residual disease (MRD) [12], since a number of clinical studies have shown that CTC detection in the peripheral blood of patients who lack any clinical or radiological signs of metastasis is related to recurrence [13] (Figure 1). Towards this goal, the molecular characterization of CTC provides critical information for the presence of therapeutic targets and resistance mechanisms that is paramount for the management of cancer patients [14].

**Figure 2:**
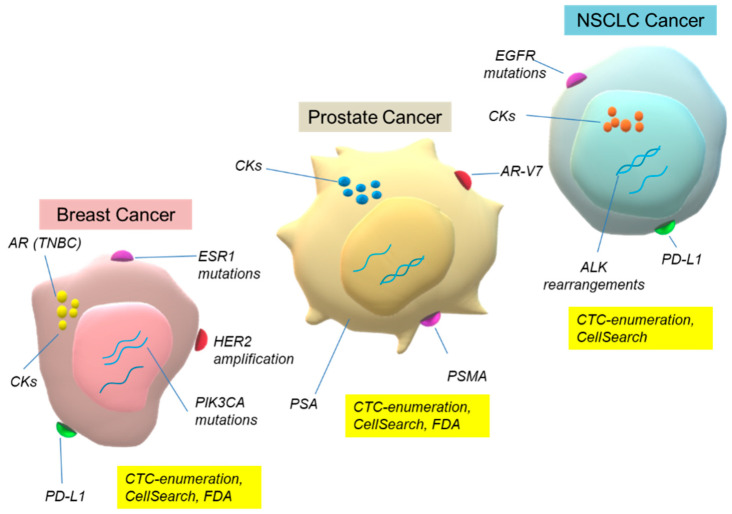
Main biomarkers detected in CTCs of breast, prostate and NSCLC.

CTC analysis is now an exponentially expanding field in almost all types of solid cancers. The main goal of this review is to summarize the latest findings over the past five years on the clinical significance of CTCs for the management of cancer patients. We focus on breast, prostate and non-small-cell lung cancer (NSCLC), highlighting the main biomarkers detected in CTCs in these three types of cancer (Figure 2). The unique potential of CTC single-cell analysis for understanding metastasis biology, and the importance of quality control and standardization, is also discussed.

## 2. CTC: Challenges for the Management of Cancer Patients

### 2.1. Breast Cancer (BrCa)

#### 2.1.1. Early Breast Cancer

##### CTC Enumeration

In a meta-analysis paper, individual patient data were collected from 21 studies in which CTC detection by CellSearch^®^ was performed in early BrCa patients treated with neo-adjuvant chemotherapy (NCT) (1574 before NCT and 1200 before surgery). It was reported that CTC-count was an independent and quantitative prognostic factor in early BrCa patients treated by NCT and that it complemented current prognostic models based on tumour characteristics and response to therapy [15]. The results of the SUCCESS A trial have shown that the detection of CTCs before and after chemotherapy using the FDA-approved CellSearch^®^ system (Menarini Silicon Biosystems, Bologna, Italy) is associated with multiple-site or bone-only first-distant disease. In this trial 3754 randomized high-risk breast cancer patients participated, 373 of whom developed metastatic disease; CTC enumeration was performed in 206 of these patients before chemotherapy and in 159 patients after chemotherapy. It was reported that, when CTCs were detected at both time points, patients showed bone-only first-distant disease and first-distant disease at multiple sites more often than patients without CTCs before and/or after chemotherapy [16].

##### CTC Biomarkers

Molecular characterization of CTCs based on gene expression, DNA methylation and DNA mutation analysis in combination with CTC count and phenotypic analysis can reveal the presence of MRD at least 4 years before the appearance of clinically detectable metastatic disease. This indicates that comprehensive liquid biopsy analysis could provide highly important information for the therapeutic management of breast cancer patients at early stages [17]. A recent study involving 1220 patients with stage I-III BrCa with more than ten-year follow-up has shown that the detection of *CK-19* mRNA-positive CTC is prognostic for early relapse, supporting investigations for novel adjuvant therapeutic approaches [18]. The prognostic significance of EMT-associated (*TWIST1*) and stem-cell (SC) transcript (*CD24*, *CD44*, *ALDH1*) quantification in EpCAM+ CTCs of early BrCa patients was examined in 100 early stage BrCa patients. This study showed that molecular detection of *TWIST1* overexpression and stem cell (*CD24*, *CD44, ALDH1*) transcripts in EpCAM-pos CTCs provided prognostic information [19].

The latest findings have proven the clinical significance of prostate biomarkers detection in triple-negative breast cancer (TNBC) patients. CTC-derived RNA isolated by 41 non-metastatic TNBC was analyzed for *PSA*, *PSMA*, *AR-FL*, and *AR-V7* expression before and after neoadjuvant chemotherapy. Although CTCs that expressed prostate-cancer-related genes were eliminated by the given therapy, *PSMA*+ CTCs identified patients at high risk of relapse, while *AR-FL*-pos CTCs, together with *AR-V7*-pos CTCs, were associated with therapeutic failure [20].

*PIK3CA* mutational status was evaluated in 13 cases of peripheral blood samples isolated by early BrCa patients before and after treatment. Before treatment, *PIK3CA* hotspot mutations were detected in plasma-ctDNA in 7/13 (53.8%) cases, and in corresponding DNAs isolated from CellSearch^®^ cartridges in 8/13 (61.5%) cases. After treatment, *PIK3CA* hotspot mutations were detected in plasma-ctDNA in 9/13 (69.2%) cases, and in DNAs isolated from CellSearch^®^ cartridges in 7/13 (53.8%) cases. *PIK3CA* hotspot mutations were detected in the large majority of DNAs isolated from CellSearch^®^ cartridges, but in a lower number of paired plasma-ctDNA samples [21].

CD47 and PD-L1 expression was also investigated on peripheral blood mononuclear cell (PBMC) cytospins from 100 early BrCa patients by triple immunofluorescence for CD47/PD-L1/cytokeratins. CD47+ and/or PD-L1+ CTCs were detected in a low proportion of early BrCa patients [22].

##### Clinical Trials

The SUCCESS clinical trial that involved 1697 early BrCa patients from the National Cancer Database and 1516 early BrCa patients showed that CTC were detected in 23.5% and 19.4% of cases, respectively. Radiotherapy after mastectomy in CTC-positive patients was associated with longer disease-free survival (DFS), and longer overall survival (OS) [23]. In 1087 early-stage, high-risk BrCa patients, CTC-counts were assessed before and 2 years after chemotherapy using the CellSearch^®^ system during the SUCCESS A trial; it was reported that detection of CTCs two years after chemotherapy was associated with decreased OS and DFS [24]. Recently, in the same trial, the correlation between the number of CTCs and the development of metastasis in high-risk early BrCa patients was studied. The presence of CTCs before and after chemotherapy could predict the presence of multiple-site or bone-only first-distant disease that may trigger intensified follow-up and further treatment [16].

#### 2.1.2. Metastatic Breast Cancer (MBC)

##### CTC Enumeration

CTC enumeration using CellSearch^®^ has been FDA approved for its prognostic significance in MBC since 2004. In this system, CTC-enrichment is based on the epithelial cell adhesion molecule (EpCAM). In a retrospective study, pooled analysis of individual patient data from 18 cohorts, including 2436 MBC patients, patients with ≥5CTCs/7.5 mL peripheral blood (PB) were classified as Stage-IV aggressive, and those with <5 CTCs/7.5 mL PB as Stage-IV indolent. Kaplan–Meier survival analysis showed that Stage-IV indolent patients had longer median OS than those classified as Stage-IV aggressive, and significantly longer OS across all disease subtypes compared to the aggressive cohort [25]. Serial CTC data derived from 469 MBC patients (2202 samples) were used to build a model to identify groups with similar CTC-trajectory patterns during treatment. Four novel prognostic groups in MBC were identified based on similarities in CTC-trajectory patterns during chemotherapy. The prognostic groups included patients with very poor outcomes (intermediate and high CTCs, 19.4%) who could benefit from more effective treatments. This novel prognostic classification approach was proposed for fine-tuning of CTC-based risk stratification strategies to guide future prospective clinical trials in MBC [26]. Another prospective study evaluated the clinical validity of combining CTC-enumeration and ctDNA analysis in 198 HER2-negative MBC patients treated by first-line chemotherapy; CTCs and ctDNA were reported to have non-overlapping detection profiles but complementary prognostic values in MBC patients [27]. In a recent study in a group of patients with MBC, antibodies against Trop-2 and CD-49f were used instead of EpCAM for CTC-enrichment in the CellSearch^®^. When the results were directly compared with the EpCAM based enrichment, it was revealed that patients with EpCAM high-expressing CTCs had worse overall and progression-free survival. However, it was suggested that, even if EpCAM low-expressing CTCs may be prognostically less relevant than EpCAM high-expressing CTCs, they could be used as a valuable tumour surrogate material and may have particular benefit if no CTCs are detected using EpCAM-dependent technologies [28].

Tumour-derived extracellular vesicles (tdEVs) also have complementary prognostic value next to CTCs. More specifically, the number of tdEVs measured by the open-source ACCEPT software that can be implemented using CellSearch^®^ images, was shown to provide prognostic information in MBC before and after one cycle of chemotherapy [29]. In May 2022, the Parsortix PC1 (Angle, UK) size-based microfluidic system for CTC enrichment was FDA-cleared for MBC. Using this system, somatic nucleotide variants (SNVs), copy number alterations (CNAs) and structural variants (SVs) were detected in CTCs, and high molecular discordance for somatic alterations between CTCs and metastases paired were identified, highlighting the intrapatient genomic differences that occur [30].

##### HER2

Numerous studies have demonstrated that HER2-positive CTCs are found in patients with HER2-negative tumours. In advanced-stage BrCa patients with HER2-negative tumours, CTC HER2-status has the potential to guide the use of anti-HER2 targeted therapy. A trial involving 264 patients with MBC showed that HER2-targeted therapy reduced the overall CTC count. This should be taken into account when CTC-status is used as an indicator for aggressive or indolent MBC [31]. In the DETECT III and IV clinical trials, 1933 patients with HER2-negative MBC were screened for participation before the initiation of a new line of therapy. CTC-enumeration was performed in the CellSearch^®^, and, in parallel, the fourth channel was used to label CTCs with an anti-HER2 antibody; the presence of ≥1 CTC/7.5 mL PB with strong HER-2 staining was associated with shorter OS [32]. Another study explored whether patients with HER2-negative tumours and HER2-positive CTC can benefit from anti-HER2 targeted therapies. Among 105 advanced-stage patients with HER2-negative breast tumours and high-risk HER2-positive CTC, those who received anti-HER2 targeted therapies had improved PFS compared to those who did not; however, anti-HER2 targeted therapy did not affect PFS in patients with low-risk CTC HER2 [33].

##### *ESR1* Mutations and Resistance to Estrogen Deprivation Therapy (EDT)

The therapeutic efficacy of hormonal therapies to target estrogen receptor (ER)-positive BrCa is limited by the acquisition of *ESR1* mutations, which confer treatment resistance to aromatase inhibitors (AIs). Constitutively active estrogen receptor-α (*ER/ESR1*) mutations have been identified in approximately one third of ER-pos MBC. Mutations, particularly in the ligand-binding domain (LBD) of *ESR1*, result in resistance to EDT in BrCa, and their detection is vital for the optimization of therapy strategies. Detection of *ESR1* mutations in CTCs and ctDNA is clinically important in comparison to primary tumours since the analysis of serial biopsies of metastatic lesions is difficult and highly invasive. Extremely sensitive and specific methodologies, such as droplet digital PCR (ddPCR) or next generation sequencing (NGS), have been developed for that reason. Numerous studies are now confirming that LB-based serial monitoring may guide the selection of precision therapeutics for women with AI-resistant ER-positive BrCa. Beyond resistance, the potential role of *ESR1* mutations in promoting metastatic disease was recently investigated. *ESR1* mutations were detected exclusively in distant, but not local, recurrences in five independent BrCa cohorts. It was reported that *ESR1*-mutant cells were associated with larger multi-cellular CTC-clusters with increased compactness compared to *ESR1*-wild type CTCs, and that CTC-clusters were enriched in patients with *ESR1*-mutated MBC. Based on these data, future therapeutic strategies for targeting *ESR1* mutant BrCa are now suggested [34]. In a prospective clinical study, 55 women with hormone receptor-positive MBC, CTCs and ctDNA were analyzed for *ESR1* mutations using multiplex-ddPCR. High-sensitivity *ESR1* sequencing from CTCs revealed mono- and oligoclonal mutations in 22% of patients that were concordant with plasma-cfDNA sequencing in 95% of cases. Detection of *ESR1* mutations was correlated both with time to metastatic relapse and duration of AI-based therapy following such recurrence and was associated with shorter PFS on AI-based therapies [35]. A low cost, highly specific, sensitive and robust assay (NAPA assay) was reported for hotspot *ESR1* mutations in CTCs and paired plasma ctDNA having concordance with drop-off ddPCR of 90.6% [36]. Recently an *ESR1* Y537N clone of CTC derived from a metastatic breast cancer patient with a TNBC, HER2-positive and estrogen receptor-positive (ER+) tumour was identified, which was characterized by the absence of mutations in the TP53 gene [37]. In a phase I trial of AZD9496, an oral selective ER degrader, single-cell CTC analysis in patients with advanced ER-POS/HER2-negative advanced breast cancer at serial time points was performed, using tandem CellSearch^®^ /DEPArray™ technologies and targeted single-cell DNA next-generation sequencing. High-quality CTC (n = 123) from 12 patients profiled by scNGS showed 100% concordance with ctDNA detection of driver *ESR1* mutations [38].

Epigenetic alterations, and more specifically *ESR1*-methylation, was recently reported as a potential biomarker for response to everolimus/exemestane treatment [39].

Beyond mutations and methylation, ER expression in the CTCs of 60 patients with metastatic breast cancer with ER-positive primary tumours at initial cancer diagnosis was evaluated in 109 longitudinal blood samples that were prospectively collected and analyzed using the CellSearch^®^ system. It was reported that only one-third of CTC-positive breast cancer patients, who were initially diagnosed with ER-positive primary tumours, harbored ER-positive CTCs at the time of metastasis, and, even in those patients, both ER-positive and ER-negative CTCs were found. The frequent presence of these ER-negative CTCs in patients with an ER-positive status in the primary tumour indicated a switch of ER phenotype or selection of minor ER-negative clones as a potential mechanism of escape from ER-targeting therapy [40].

##### *PIK3CA* Mutations

*PIK3CA* mutations have been detected in primary tissues, ctDNA and CTCs. A specific drug (Alpelisib PIQRAY, Novartis) is now indicated in combination with fulvestrant for the treatment of post-menopausal women with HR-positive, HER2-negative, *PIK3CA*-mutated, advanced or MBC, following progression on or after an endocrine-based regimen. Three FDA-approved *PIK3CA* mutation companion diagnostic tests are now available for primary tissues or plasma ctDNA. A direct comparison study for *PIK3CA* hotspot mutations in CTCs and paired ctDNA isolated through the same blood draw revealed that *PIK3CA* hotspot mutations were present at high frequencies in CTCs and paired plasma-ctDNA [21]. *PIK3CA* mutational status significantly changed after therapeutic intervention, and CTCs and plasma-ctDNA provided complementary information [21].

##### Biomarkers for Immunotherapy

Expression of programmed death-ligand 1 (PD-L1) on CTCs or circulating immune effector cells could provide insights into the selection of patients for immune checkpoint inhibition (ICI). The results of a dedicated prospective clinical trial that investigated the clinicopathological correlations and prognostic value of PD-L1(+)-CTCs in patients with MBC showed that PD-L1(+)-CTCs were significantly associated with PFS, while tissue PD-L1 expression was not [41]. The evaluation of PD-L1 expression in CTC and platelets in MBC, revealed its potential role in predicting which patients should receive ICI [42]. In MBC, CD47^high^ and/or PD-L1^high^ CTCs are associated with disease progression and shorter PFS and independently predict increased risk of relapse and death [22].

##### CTCs Biomarkers

It was recently reported that CXCR4, JUNB and TYROBP are overexpressed in CTCs, but only JUNB expression was associated with poor prognosis [43]. Another study that investigated the prognostic relevance of single CSC+/partial-EMT+ CTCs in MBC before and after first-line chemotherapy showed that they represent a chemoresistant subpopulation, which independently predict unfavorable outcome in MBC; efficient targeting of these CTCs could potentially increase patient survival [44]. The CSC+/partial-EMT+ phenotype also represents the most frequent subset of CTC-positive MBC patients, which causes reduced survival rates during Eribulin treatment [45]. TLR4 and pSTAT3 are key players in cancer inflammation and immune evasion; in MBC TLR4-pos CTCs correlated with a high risk of disease progression [46]. In MBC, RT-qPCR analysis in CTC-derived RNA for stem-cell markers (*CD24*, *CD44*, *ALDH1*), *TWIST1*, *ESR1*, *PGR*, *HER2*, *EGFR* and *CK-19* revealed that the combined gene expression of *CK-19*(+), *CD44^high^/CD24^low^*, *ALDH1^high^/CD24^low^* and *HER2* overexpression was correlated with OS [47]. A 17-gene digital RNA signature of breast CTC-derived transcripts was reported to enable early monitoring of response with high sensitivity. In a prospective cohort of localized BrCa, an elevated CTC score after three cycles of neoadjuvant therapy was found to be associated with MRD at surgery. In a second prospective cohort with MBC, the baseline CTC score was correlated with OS, as was a persistent CTC signal, after 4 weeks of treatment [48]. Comprehensive molecular analysis at the gene expression, DNA mutation, and DNA methylation levels based on in vivo CTC isolation was proposed as a powerful approach for molecular diagnostic applications based on CTCs. In this study, CTC-derived RNA was analyzed for *CK8*, *CK18*, *CK19*, *ERBB2*, *TWIST1*, *VEGF*, *ESR1*, *PR*, *EGFR*, *CD44*, *CD24*, *ALDH1*, *VIM*, and *CDH2* and *B2M* (reference gene) expression, while CTC-derived DNA was analyzed for *PIK3CA* mutations and *ESR1* methylation [49]. In a recent study, using RT-qPCR, two panels of transcripts related to the presence of CTCs (Panel 1: *CK19*, *EpCAM*, *SCGB2A2* and Panel 2: *EMP2*, *SLC6A8*, *HJURP*, *MAL2*, *PPIC* and *CCNE2*), in two cohorts of breast cancer patients (metastatic and early), were evaluated. The findings indicated that Panel 2 markers that were not linked to an epithelial phenotype also provided important prognostic information [50].

Moreover, a combination of multiple liquid biopsy analytes, such as matched CTC-mRNA, CTC-gDNA EV-mRNA, and cfDNA, evaluated in a pilot study involving HR-pos, and HER2-neg MBC patients, was shown to have additional benefits for OS prediction [51]. miRNAs are also very important biomarkers in MBC, mainly through their role in epigenetic modification [52]. Combined increased levels of miR-200s and CTC count have been shown to have superior prognostic significance for the clinical management of MBC patients [53].

##### Clinical Trials

The main clinical studies investigating the clinical utility of CTCs were recently updated in an overview of clinical trials and treatments based on CTC-count or CTC variations, and treatments based on the molecular characteristics of CTCs [54]. The efficacy of trastuzumab-emtansine was evaluated in 154 HER2-negative MBC patients with HER2-positive CTC. Patients were eligible for treatment only if they had more than one HER2amp CTC. It is important to point out that CTCs with HER2-amplification were detected in a small group of HER2-negative MBC patients and that partial response was observed in one patient after administration of trastuzumab-emtansine [55]. Using existing datasets, machine learning was used to develop a classifier for CTC prognostic simulation to identify patients with MBC likely to have ≥5CTCs/7.5 mL blood. The prognostic impact of this simulation was similar to patients with actual CTC-enumeration [56]. In the STIC-CTC clinical trial, 755 women diagnosed with HR-positive, HER2-negative MBC were randomized to a clinician-driven choice of first-line treatment or a CTC count-driven first-line treatment choice. A total of 377 patients were allocated to the CTC arm and 378 patients were allocated to the standard arm, and 138 (37%) and 103 (27%) received chemotherapy, respectively. In the discordant clinical low CTC high subgroup, PFS was significantly higher in the CTC arm, indicating that the CTC count could be a reliable biomarker method for guiding the choice between chemotherapy and endocrine therapy as the first-line treatment in HR-positive, HER2-negative MBC [57]. In a multicenter clinical trial, peripheral blood samples from 216 patients with metastatic breast cancer (MBC) and 205 healthy volunteers were subjected to CTC enrichment using the Parsortix device. CTCs were enumerated from each participant by cytologic evaluation of Wright–Giemsa-stained slides and their detection was performed using molecular profiling by qRT-PCR, RNA sequencing, or cytogenetic analysis of HER2 amplification by FISH. The study demonstrated that the Parsortix^®^ PC1 system could effectively capture and harvest CTCs from the peripheral blood of MBC patients and that the harvested cells could be evaluated using orthogonal methodologies, such as gene expression and/or fluorescence in situ hybridization (FISH) [58].

A summary of recent CTC-based studies in breast cancer is presented in Table 1.

### 2.2. Prostate Cancer (PCa)

#### 2.2.1. Non-Metastatic

High-risk non-metastatic PCa has the potential to progress into lethal disease since it remains unclear which treatment offers the best results. However, diagnosis and prognosis of non-metastatic prostate cancer patients could gain another perspective by applying molecular tests to peripheral blood [59]. Moreover, the detection and molecular characterization of CTCs can lead to a promising strategy for monitoring MRD or relapses following local therapy. A recent study compared three innovative technologies for CTC enumeration in 131 high-risk patients with PCa, before and after radiotherapy, combined with androgen deprivation: CTC-enumeration (CellSearch^®^), the dual fluoro-EPISPOT assay, and the in vivo CellCollector^®^ technology. The concordance among methods was only 23%, but the cumulative positivity rate was 79%, suggesting that combining different CTC-assays improved CTC detection rates in patients with non-metastatic high-risk PCa before and after treatment [60]. In vivo CTC enrichment in high-risk non-metastatic PCa patients has the potential to detect CTC at a higher efficiency compared to CellSearch^®^ [61]. Joose et al. investigated whether prostate biopsy was associated with release of prostatic tumour cells into the circulation using CellSearch^®^ before and within 30 min after performing prostate biopsy from 115 men with increased serum PSA. The results showed that CTC-counts increased significantly after biopsy and that this biopsy-related increase of CTC-counts was significantly correlated with worse PFS [62]. In patients undergoing radical prostatectomy for oligometastatic PCa, CTC-counts were a prognostic factor and were more closely associated with prognosis than other biomarkers commonly used in daily clinical practice [63].

#### 2.2.2. Metastatic Prostate Cancer (mPCa)

##### CTC Enumeration

CellSearch^®^ has been FDA-approved for its prognostic significance in mPCa since 2007. Recently a consensus statement on circulating biomarkers in advanced PCa included the quantitation and characterization of CTCs and cell-free nucleic acids for therapeutic monitoring and as prognostic and predictive biomarkers [64]. When the clinical significance of any increase in CTC-counts was examined as an indicator of progression in 511 PCa patients with low levels of CTCs before treatment (<5/7.5 mL), it was shown that increasing CTCs during the first 12 weeks of treatment was independently associated with worse OS in patients treated with abiraterone or chemotherapy [65]. CTC-enriched and CD45-depleted fractions isolated from the CellSearch^®^ cartridges using CellCelector (ALS, Jena, Germany) can be further characterized with multicolor flow cytometry. The phenotype of panCK+ CXCR4+ CTCs has prognostic potential in patients treated by radiotherapy. In a cohort of 24 mPC patients the total CTC count dropped after radiotherapy, while a subpopulation of CTC expressing CXCR4 remained stable up to three months, indicating that these cells preferentially survive during local radiotherapy [66].

##### *AR-V7* 

Many clinical studies have shown that in mCRPC, patients expressing *AR-V7* in CTCs have a greater benefit from taxane chemotherapy compared to hormonal therapies. *AR-V7* is a highly promising LB predictive biomarker showing primary or acquired resistance to novel AR signaling inhibitors. Its detection in CTC using the AdnaTest mRNA or Epic nuclear protein assays represents the first analytically and prospectively clinically validated LB-based assays that may inform treatment decisions in men with mCRPC [67]. The expression profile of *AR-FL*, *AR-V7*, *AR-567es*, and *CK-19* transcripts in EpCAM-pos CTCs, and paired plasma-derived EVs from 62 mCRPC patients have shown remarkable heterogeneity [68]. In another study involving 227 peripheral blood samples from 181 mCRPC patients, CTC+/*AR-V7*+ samples had higher CTC-counts and biopsy AR-V7 protein expression than CTC+/*AR-V7*- samples; however, both CTC- and CTC+/*AR-V7*- patients had detectable AR-V7 protein in tissue biopsies [69]. Use of the nuclear-localized AR-V7 CTC test to inform treatment choice can improve patient outcomes relative to decisions based solely on standard-of-care measures [70]. Beyond *AR-V7*, a transcriptional profile detectable in CTCs can serve as an independent prognostic marker in patients with mPCa and can be used to identify the emergence of resistance mechanisms [71].

##### PD-L1

According to a systematic literature review, in 101/159 (64%) liquid biopsies of PC patients, a variable number of PD-L1+CTCs was detected, while nuclear *PD-L1* expression in CTCs was occasionally associated with worse prognosis. PD-L1 status was discordant for primary vs. metastatic tissue biopsies and CTCs vs. corresponding tumour tissues [72].

##### Detection of Biomarkers in CTCs

The integration of different isolation and detection systems, such as DLA, AdnaTest and NanoString nCounter technology could be very important to increase the sensitivity of CTC detection in mPC patients. RNA profiling of DLA-enriched CTCs could provide important information about the aggressiveness of the CTC phenotype at the time that a patient is experiencing a disease relapse and the level of serum prostate specific antigen (PSA) is relatively low [73]. A comparison study between gene expression and DNA methylation markers in CTCs and corresponding plasma-derived exosomes in mCRPC showed a higher positivity rate in gene expression in EpCAM-positive CTCs, whereas DNA methylation was detected both in CTC and exosomes. It is important to mention that some of the markers tested correlated with OS [74]. Gene expression profiling of 14 genes in CTCs isolated in vivo from 108 high-risk PCa patients revealed high heterogeneity, while direct comparison with CTC-enumeration (CellSearch^®^), PSA-EPISPOT, and immunofluorescence showed a relatively low concordance between these different methodologies [75].

##### Clinical Trials

In mCRPC clinical research and practice, measures of response that are clinically meaningful and occur early are an unmet need. Using individual patient data, a recent study explored CTC and PSA response end points in five prospective randomized phase III trials that enrolled in total 6081 patients (COU-AA-301, AFFIRM, ELM-PC-5, ELM-PC-4, and COMET-1 clinical trials. Gov identifiers: NCT00638690, NCT00974311, NCT01193257, NCT01193244, and NCT01605227, respectively). Analysis of results showed that CTCs were detected at baseline but not at 13 weeks, and that a reduction in CTC-counts had the highest discriminatory power for OS [76]. Another multicenter prospective blinded study of patients with poor-risk mCRPC, assessing *AR-V7* status in CTC from 118 mCRPC patients, showed that pretreatment CTC-*AR-V7* status was independently associated with worse PFS and OS and a low probability of confirmed PSA responses (0% to 11%) during treatment with abiraterone or enzalutamide [77].

A summary of recent CTC-based studies in prostate cancer is presented in Table 2.

### 2.3. NSCLC

#### 2.3.1. Early Stage

NSCLC patients positive for CTCs in peripheral blood at the time of surgery have a higher risk of recurrence. In early-stage NSCLC, the TRACERx study investigated pulmonary venous CTC (PV-CTC) counts using CellSearch^®^; it was reported that these CTCs represented subclones that were responsible for relapse and remained an independent predictor of relapse in multivariate analysis adjusted for tumour stage. Moreover, genomic profiling of single PV-CTCs collected at surgery revealed higher mutation overlap, with metastasis detected 10 months later than with the primary tumour [78].

#### 2.3.2. Advanced NSCLC

##### CTC Enumeration

CTC-enumeration has been assessed for prognostication of patients with advanced NSCLC by undertaking a pooled analysis of individual patient data by nine European NSCLC CTC centers. A total of 7/9 eligible centers provided data for 550 patients with prognostic information for OS. CTC-counts of ≥2 and ≥ 5/7.5 mL were associated with reduced PFS and OS, respectively [79]. In 16 advanced NSCLC patients, using a single tube approach, EpCAM-high CTC, EpCAM-low CTC, tdEVs and ctDNA were evaluated as prognostic biomarkers for OS. The presence of EpCAM-high CTC and elevated levels of tdEV and ctDNA was associated with poor OS, while the presence of EpCAM-low CTC was not [80].

##### PD-L1

In a prospective study, CTC enrichment (Parsortix, Angle UK) was used to assess PD-L1 expression in CTCs from 127 samples from NSCLC, 97 of which were also analyzed by CellSearch^®^. Using Parsortix, 61% of samples were CTC-positive, while CTCs were detected in 32% using CellSearch^®^. In all patients, upon disease progression, an increase in PD-L1+CTCs was observed, while in responding patients there was no change or decrease in PD-L1+CTCs [81]. An evaluation of PD-L1 expression in CTCs (CellSearch^®^) from 54 advanced NSCLC patients showed that the presence of PD-L1(+) CTCs was associated with poor prognosis [82]. CTCs can also be used to monitor dynamic changes of PD-L1 during radiation therapy, which is potentially prognostic of response to treatment [83]. The evaluation of PD-L1 and EMT marker expression in the CTCs of 30 NSCLC patients that underwent surgery showed that these markers were expressed at significantly higher proportions in CTCs than in patient-matched NSCLC tissues. PD-L1pos/EMTpos CTCs were also associated with significantly poorer survival after curative surgery [84]. The differential expression of PD-L1 and Ki67 on CTCs can also provide further predictive information in advanced NSCLC patients treated with pembrolizumab. Changes in a PD-L1 low subpopulation at an early phase of treatment were importantly related to disease control or resistance to pembrolizumab immunotherapy [85]. High expression of *PD-L1* at PD of NSCLC patients during osimertinib therapy indicates that immunotherapy could be effective in NSCLC patients positive for *EGFR* mutations that develop resistance to osimertinib [86].

##### Anaplastic Lymphoma Kinase (ALK) Rearrangements

Patients with ALK-rearranged NSCLC inevitably develop resistance to ALK inhibitors. Resistance mutations have been investigated in CTCs isolated at the single-cell level from patients at disease progression on crizotinib or lorlatinib. Multiple mutations in various genes in ALK-independent pathways RTK-KRAS (EGFR, KRAS, BRAF genes) and TP53 pathways were predominantly identified in CTCs of crizotinib-resistant patients [87]. Gatekeeper mutations were identified in only 50% of the cases at resistance to ALK inhibitors. An in-depth investigation of copy number alteration (CNA) heterogeneity in phenotypically characterized CTCs at resistance to ALK-TKIs in ALK-positive NSCLC at the single cell level, revealed that, in FACS-isolated CTCs, 37% were ALK+/cytokeratins-, 56% were ALK-/cytokeratins+, and only 5% were ALK+/cytokeratins+. CTC sequencing revealed wide CNA heterogeneity, while high levels of CIN in CTCs indicated resistance to ALK-TKIs. ALK-rearranged CTCs showed EMT characteristics potentially contributing to ALK-TKI resistance [88].

##### EGFR–TKIs-Osimertinib

Osimertinib is given as a second-line treatment in EGFR mutant NSCLC patients. A multiparameter liquid biopsy analysis in plasma cfDNA and paired CTCs in NSCLC patients under osimertinib treatment was performed at different time points at the DNA-mutation, DNA-methylation and gene expression levels. Molecular methodologies used to detect molecular targets associated with TKI treatment should be further validated in large prospective studies [89]. Based on this, recently, Crystal digital PCR (STILLA, France) was used to detect and quantify %MAFs of *EGFR* mutations in ctDNA and paired CTCs. A strong correlation between results in ctDNA with the corresponding primary tissue revealed a high heterogeneity in *EGFR* mutations, while a small number of samples were found to be positive for EGFR mutations in CTC fractions [90]. DNA methylation represents a mechanism of therapy resistance in NSCLC [91]. In a recent study, DNA methylation of nine selected genes was examined in plasma-cfDNA; paired CTCs revealed different methylation patterns, indicating that CTCs and cfDNA give complementary information [92]. A high prevalence of VIM-positive CTCs from 30 NSCLC patients under osimertinib therapy suggested a dynamic role of EMT [86].

A summary of recent CTC-based studies in NSCLC is presented in Table 3.

## 3. CTC: Ongoing Clinical Trials, Novel Dimensions and Future Challenges

The clinical utility of CTC-enumeration and molecular characterization, as shown so far, has led to the design of clinical trials that include CTC-analysis. More specifically, a recent search has revealed that there are: (a) 396 clinical trials including CTC analysis in breast cancer (https://clinicaltrials.gov/ct2/results?term=CTCs&cond=breast+cancer, accessed on 2 March 2023), 183/396 are completed, 108/396 are still ongoing recruiting patients, while 49/396 are of unknown status, 10/396 were withdrawn and 46/396 were suspended or terminated, (b) 313 clinical trials that include CTC analysis were registered to the clinical trials registry, in prostate cancer (https://clinicaltrials.gov/ct2/results?term=CTCs&cond=prostate+cancer, accessed on 2 March 2023), 128/313 of which are already completed, 102/313 are still ongoing, while 26/313 are of unknown status, 18/313 were withdrawn and 39/313 were suspended or terminated, (c) 225 clinical trials that include CTC analysis were registered to the clinical trials registry, in NSCLC (https://clinicaltrials.gov/ct2/results?term=CTCs&cond=NSCLC, accessed on 2 March 2023), 87/225 of which are already completed, 54/225 are still ongoing, while 48/225 are of unknown status, 7/225 were withdrawn and 29/225 were suspended or terminated.

A few years ago, the formation of cancer cell lines derived from CTC was successful based on CTC isolation from a metastatic colon cancer patient at different time points. Detailed characterization of these CTC-derived cell lines has shown a deregulation of different genes that promote drug resistance, xenobiotic and energy metabolism, stem cell properties and plasticity [93]. Moreover, CTC-derived xenograft (CDX) models and CTC-derived ex vivo cultures were developed and used to explore tumour-initiating cells and to uncover new therapeutic targets [94]. CTC-derived organoids have the potential to provide a powerful tool for personalized cancer therapy but are restrained by low CTC numbers. CTC enrichment using diagnostic leukapheresis could provide the high number of CTCs required for the formation of CTC-derived organoids. Using DLA, successful organoid cultures containing significantly higher CTC numbers at initiation and a substantial tumour heterogeneity in CTCs using single-cell DNA sequencing was observed [95].

Detection of CTC-clusters has been found to be connected with increased metastatic potential and specific changes in DNA methylation; these changes were related to stemness and metastasis, indicating that cluster-targeting compounds could be tested as potential suppressors of cancer spread [96]. CTC clusters are mainly detected during advanced disease stages, but a recent study has highlighted the presence of CTC clusters at early disease stages. The early BrCa detection of CTC-clusters prior to surgical treatment indicates that intervention with anti-cluster therapeutics could be tested even in the early stages [97]. Dissemination of CTC-clusters could be seen as an early event in breast cancer since CTC-clusters are more frequent in early BrCa than in MBC patients [98].

CTC analysis at the single-cell resolution level provides unique insights into tumour heterogeneity that cannot be revealed by ctDNA analysis since it can address many different cellular components that can influence tumour heterogeneity [99]. Analysis of CTCs and CTC-clusters at the single-cell level provides crucial information on metastatic mechanisms and the genomic alterations responsible for drug resistance, empowering treatment and the management of cancer [100].

## 4. Conclusions—Future Perspectives

The prognostic relevance of CTCs has been proven in many types of solid cancer and changes in CTC-counts during therapy can predict treatment response. The clinical utility of CTC-enumeration and molecular characterization, as shown so far, has led to the design of clinical trials that include CTC-analysis, as mentioned above. There is now an urgent need for standardization and quality control of all steps of CTC-analysis. Comparison data between different technologies using the same samples show low concordance. Standard cell lines for quality control of CTC-analysis and ring studies are urgently needed. Towards this goal, several organizations, such as the European Liquid Biopsy Society (ELBS), the International Liquid Biopsy Standardization Alliance (ILSA), the International Society of Liquid Biopsy (ISLB), the International Federation of Clinical Chemistry (IFCC), and the European Federation of Laboratory Medicine (EFLM) have focused on developing reliable and sustainable diagnostic and prognostic tools that will benefit patient health management and wellness. The CANCER-ID consortium, still in operation in the context of ELBS, has focused on assessment of the technical validity of CTC detection methods in a European multi-center setting, aiming to establish guidelines and definition of minimal performance qualification requirements prior to clinical validation of technologies [101]. ILSA is comprised of numerous international organizations and foundations that focus on liquid biopsy and aim to establish oncology practice to support clinical decision making and regulatory considerations. Recently, a white paper was published on the independent liquid biopsy- and standardization-based programs engaged with ILSA, their objectives and progress to date, and the tools and resources each is developing to contribute to the field [102]. Another important dimension is the health economic perspective of liquid biopsies in cancer management. Beyond studies on the clinical utility of CTCs, translation of these tests into clinical practice also requires systematic assessment of the health economic benefits [103].

In conclusion, CTC-analysis provides important information for the management of cancer patients and is soon to be integrated into clinical practice based on results from interventional studies.

## Figures and Tables

**Table 1:** Overview of clinical studies in CTCs on breast cancer (2018–2022).

CTC Enrichment	CTC Detection	Biomarkers Tested	Patients (Positivity)	Clinical Significance	Ref.
**Early breast cancer**					
CellSearch^®^	IF: CTC enumeration	panCK-, DAPI, CD45	1574: ≥ 5 CTCs (25.2%)	Yes: DFS, *p* < 0.001 Yes: OS, *p* < 0.001	[15]
EpCAM-based immunomagnetic enrichment	RT-qPCR	*CK-19*	1220 (39.6%)	Yes: OS, *p* < 0.001	[18]
EpCAM-based immunomagnetic enrichment	Multiplex RT-qPCR	*CD24*, *CD44*, *ALDH1, TWIST1*	100: *TWIST1* (19%), *CD24^−/low^/CD44^high^* (15%), *CD24^−/low^/ALDH1^high^* (9%)	Yes: DFS, *p* < 0.001 Yes: OS, *p* < 0.001	[19]
CellSearch/DNA isolation from cartridges	Droplet digital PCR (ddPCR)	*PIK3CA* hotspot mutations	56 (66.1%)	no information	[21]
Ficoll-hypaque density gradient	Triple IF	CD47/PD-L1/Cytokeratins	100 CK+ (15%) CD47 (8%) PDL1 (2%)	PFS: n.s O.S: n.s	[22]
CellSearch^®^	IF: CTC enumeration	panCK-, DAPI, CD45	3213: ≥5 CTCs (21.6%)	Yes: OS, *p* < 0.001	[23]
CellSearch^®^	IF: CTC enumeration	panCK-, DAPI, CD45	1087: ≥5 CTCs (18.2%)	Yes: DFS, *p* < 0.001 Yes: OS, *p* < 0.001	[24]
**Metastatic breast cancer (MBC)**					
CellSearch/DNA isolation from cartridges	ddPCR	*PIK3CA* hotspot Mutations	22 (81.5%)	no information	[21]
Ficoll-hypaque density gradient	Triple IF	CD47/PD-L1/ Cytokeratins	98: CK+ (22.4%) CD47 (14.8%) PDL1 (7.4%)	Yes: PFS, *p* = 0.010 No: OS, 0.184	[22]
CellSearch^®^	IF: CTC enumeration	panCK-, DAPI, CD45	1944: ≥ 5 CTCs (46.9%)	Yes: OS, *p* < 0.0001	[25]
CellSearch^®^	IF: CTC enumeration	panCK-, DAPI, CD45	469: ≥ 5 CTCs (53.3%)	Yes: DFS, *p* < 0.001 Yes: OS, *p* < 0.001	[26]
CellSearch^®^	IF	CD45 CK8,18,19	510: ≥1 CTC (72%), ≥5 CTC (49%)	Yes:PFS, *p* < 0.0001 YES: OS, *p* < 0.0001	[27]
	Τargeted NGS	54 genes for mutation analysis	510 (28.8%)		
CellSearch^®^	IF: CTC enumeration	panCK-, DAPI, CD45 HER2	264: ≥5 CTCs; HER2 therapy (17.9%), New HER2 therapy (46.7%), and No HER2 therapy (46.2%)	no information	[31]
CellSearch^®^	IF: CTC enumeration	panCK-, DAPI, CD45	1933: ≥1 CTC (63.0%)	Yes:OS, *p* = 0.013	[32]
CellSearch^®^	IF: CTC enumeration	panCK-, DAPI, CD45	105: ≥5 CTCs (59.0%)	Yes: PFS, *p* = 0.008	[33]
CTC-iChip	ddPCR	*ESR1* mutations	55 (22%)	Yes: PFS, *p* = 0.0006	[35]
EpCAM-based immunomagnetic enrichment	MSP	*ESR1* methylation	112 (23.3%)	Yes: PFS, *p* = 0.009 Yes: OS, *p* = 0.028	[39]
CellSearch^®^	IF: CTC enumeration	panCK-, DAPI, CD45 PD-L1	72: ≥5 CTCs (79.2%), PD-L1(+)-CTCs (36.1%)	Yes: PFS, *p* = 0.03 Yes: OS, *p* = 0.05	[41]
CellSearch^®^	IF: CTC enumeration	panCK-, DAPI, CD45 PD-L1	52: ≥1% PD-L1(+)-CTC (40%)	no information	[42]
Ficoll-hypaque density gradient	Confocal laser scanning microscopy	JUNB, YWHAB, TYROBP, NFYA, and PRDX1	100: JUNB (65%), TYROBP (75%), NFYA (14.3%), and PRDX (12.5%)	Yes: PFS, *p* = 0.015 Yes: OS, *p* = 0.002	[43]
Ficoll-hypaque density gradient	Triple IF	Cytokeratins, ALDH1, and TWIST1	130 (27.7%)	Yes: PFS, *p* = 0.024 Yes: OS, *p* = 0.020	[44]
EpCAM-based immunomagnetic enrichment	RT-qPCR	*CD24*, *CD44*, *ALDH1, TWIST1*, *ESR1*, *PGR*, *HER2*, *EGFR*, *CK-19*	46: *TWIST1* (2.2%), *CD24* (45.7%), *CD44* (26.1%), *ALDH1* (8.7%), *ESR1* (13.0%), *HER2* (17.4%), *CK19* (21.7%), *EGFR* (0%)	Yes: OS, *p* = 0.001	[47]
In vivo CellCollector	Multiplex Real-time PCR and ddPCR	*CK8*, *CK18*, *CK19*, *ERBB2, TWIST1*, *VEGF*, *ESR1*, *PR*, *EGFR*, *CD44*, *CD24*, *ALDH1*, *VIM*, and *CDH2* *PIK3CA* and *ESR1 mutations*	42: At least one gene before therapy (54.8%) 13: At least one gene after therapy(61.5%)	no information	[49]
CellSearch^®^	IF: CTC enumeration	*HER2*-positive *CTC*	154: ≥1 CTC (78.7%), ≥5 CTC (57.3%), ≥1 HER2_amp_ CTC (9.1%)	No: PFS No: OS	[55]
CellSearch^®^	IF: CTC enumeration	panCK-, DAPI, CD45	391 CTC-driven choice arm (36.6%)	Yes: PFS, *p* < 0.05 No: OS, *p* > 0.05	[57]
Parsortix device	RT-qPCR, RNA-seq, FISH	*GYPA*, *PTPRC*, *EpCAM*, *KRT19*, *ERBB2*, *TWIST1*, *SNAI2*	194 ≥ 5 CTC (22.7%) Gene expression (47.9%)	no information	[58]

**Table 2:** Overview of clinical studies in CTCs in prostate cancer (2018–2022).

CTC Enrichment	CTC Detection	Biomarkers Tested	Positivity (%)	Clinical Significance	Ref.
CellSearch^®^	IF: CTC enumeration	panCK-, DAPI, CD45	177 ≥ 1 CTCs (14%)	no information	[60]
EPISPOT	Detection of secreted proteins	PSA/FGF2	192 ≥ 1 CTCs (42%)		
In-vivo (CellCollector)	IF	panCK-, DAPI, CD45	190 ≥ 1 CTCs (48%)		
In-vivo (CellCollector)	IF	panCK-, DAPI, CD45	51 (39.2%)	no information	[61]
CellSearch^®^	IF: CTC enumeration	panCK-, DAPI, CD45	51 (18.6%)		
CellSearch^®^	IF: CTC enumeration	panCK-, DAPI, CD45	105 ≥ 1 CTCs(20%)	Yes: PFS, *p* = 0.0021	[62]
CellSearch^®^	IF: CTC enumeration	panCK-, DAPI, CD45	511 (49.7%)	Yes: OS, *p* < 0.001	[64]
EpCAM-based immunomagnetic enrichment	Multiplex RT-qPCR	*AR-FL*, *AR-V7*, *AR-567es* and *AR-total*	69: *AR-FL* (92.3%), *AR-V7* (49.3%), *AR-567es* (23.2%), *AR-total* (89.9%)	no information	[68]
Positive immunomagnetic enrichment (AdnaTest)	Multiplex RT-qPCR	*PSMA*, *PSA*, *EGFR*	227: CTC+/*AR-7+* (35%)	Yes: OS, *p* = 0.02	[69]
All cells analyzed (Epic technology)	IF	cytokeratins, CD45, and AR-V7	255 pts blinded to AR-V7 status	Yes: OS, *p* = 0.041	[70]
EpCAM-based immunomagnetic enrichment	Multiplex RT-qPCR	*CK-8*, *CK-18*, *TWIST1*, *PSMA*, *AR-FL*, *AR-V7*, *AR-567* and *PD-L1*	57 (85.9%)	Yes: OS, *p* = 0.001	[74]
	IF: CTC enumeration	panCK-, DAPI, CD45	57: ≥ 1 CTC (85.9%)		
In-vivo (CellCollector)	Multiplex RT-qPCR	*KRT19*, *EpCAM*, *CDH1*, *HMBS*, *PSCA*, *ALDH1A1*, *PROM1*, *HPRT1*, *TWIST1*, *VIM*, *CDH2*, *B2M*, *PLS3*, and *PSA*	74 (70.5%)	no information	[75]

**Table 3:** Overview of clinical studies in CTCs on NSCLC (2018–2022).

CTC Enrichment	CTC Detection	Biomarkers Tested	Patients Positivity %	Clinical Significance	Ref.
CellSearch^®^	IF: CTC enumeration	panCK-, DAPI, CD45	100 (48%)	Yes: PFS, *p* = 0.019	[78]
CellSearch^®^	IF: CTC enumeration	panCK-, DAPI, CD45	97: ≥2 EpCAM^high^ CTC (21%), ≥2 EpCAM^low^ CTC (15%)	Yes: OS, *p* = 0.014	[80]
CellSearch^®^	IF: CTC enumeration	panCK-, DAPI, CD45 PD-L1	54: CTCs(43.4%),PD-L1(+) CTCs (9.4%)	Yes: PFS, *p* = 0.006 Yes: OS, *p* = 0.002	[82]
Microfluidic chip	IF	PD-L1 CK+/CD45−/DAPI+	38: PD-L1(+) CTCs (69.4%)	Yes: PFS, *p* = 0.017	[83]
	qPCR	PD-L1	38 (36.4%)		
Size-based enrichment (Parsortix)	Multiplex RT-qPCR	*CK-8*, *CK-18*, *CK-19)*, *VIM*, *TWIST-1*, *AXL*, *ALDH-1*, *PD-L1 PIM-1*	30: epithelial markers (37%), mesenchymal/EMT markers (65.4%), stem cell marker (29.6%)	no information	[86]
RosetteSep/DEPArray	Target NGS	ALK mutations	14 (64.3%)	no information	[87]
FACS/DEPArray	IF	ALK/ cytokeratins/ CD45/	82 CTCs: ALK^+^/cytokeratins^-^(37%),ALK^-^/cytokeratins^+^ (56%), ALK^+^/cytokeratins^+^(5%)	no information	[88]
Size-based enrichment (Parsortix)	ddPCR	EGFR mutations	64 (17.2%)	no information	[90]
